# Possible Interbreeding in Late Italian Neanderthals? New Data from the Mezzena Jaw (Monti Lessini, Verona, Italy)

**DOI:** 10.1371/journal.pone.0059781

**Published:** 2013-03-27

**Authors:** Silvana Condemi, Aurélien Mounier, Paolo Giunti, Martina Lari, David Caramelli, Laura Longo

**Affiliations:** 1 UMR 7268 CNRS/Aix-Marseille Université/EFS ADES - Anthropologie bioculturelle, Droit, Ethique et Santé Faculté de Médecine - Secteur Nord Aix-Marseille Université, Marseille, France; 2 The Leverhulme Centre for Human Evolutionary Studies Biological, Anthropology Division, Department of Archaeology and Anthropology, University of Cambridge, Cambridge, United Kingdom; 3 Istituto Italiano di Preistoria e Protostoria, Firenze, Italy; 4 Università di Firenze, Dipartimento di Biologia Evoluzionistica, Laboratorio di Antropologia,Unità di Antropologia Molecolare/Paleogenetica, Firenze, Italy; 5 Musei Civici Fiorentini, Firenze, Italy; University of Kansas, United States of America

## Abstract

In this article we examine the mandible of Riparo Mezzena a Middle Paleolithic rockshelter in the Monti Lessini (NE Italy, Verona) found in 1957 in association with Charentian Mousterian lithic assemblages. Mitochondrial DNA analysis performed on this jaw and on other cranial fragments found at the same stratigraphic level has led to the identification of the only genetically typed Neanderthal of the Italian peninsula and has confirmed through direct dating that it belongs to a late Neanderthal. Our aim here is to re-evaluate the taxonomic affinities of the Mezzena mandible in a wide comparative framework using both comparative morphology and geometric morphometrics. The comparative sample includes mid-Pleistocene fossils, Neanderthals and anatomically modern humans. This study of the Mezzena jaw shows that the chin region is similar to that of other late Neanderthals which display a much more modern morphology with an incipient mental trigone (e.g. Spy 1, La Ferrassie, Saint-Césaire). In our view, this change in morphology among late Neanderthals supports the hypothesis of anatomical change of late Neanderthals and the hypothesis of a certain degree of interbreeding with AMHs that, as the dating shows, was already present in the European territory. Our observations on the chin of the Mezzena mandible lead us to support a non abrupt phylogenetic transition for this period in Europe.

## Introduction

One of the most frequently debated questions in paleoanthropology concerns the arrival in Europe of our species, *Homo sapiens*, anatomically modern humans (AMHs), and the fate of the humans who lived in this territory prior to their arrival, the Neanderthals.

For many decades there were two different responses to this question: according to one point of view the Neanderthals did not really disappear, but were incorporated into the new incoming modern humans. Authors who support this hypothesis have argued that there was a change in morphology of late Neanderthals [1]–[3] and have interpreted certain anatomic features observed among early AMHs in Europe as a result of a continuity with Neanderthals [4], [5]. A certain degree of continuity has also been supported by archaeologists who have identified much more complex forms of behavior among Neanderthals than was previously acknowledged [6], [7]. European Neanderthals have been considered to be not only the producers of Mousterian assemblages but also the makers of the later so-called "transitional assemblages" (Chatelperronian, Uluzzian, Bohunician, Szeletian) [8], [9], either by internal modification [7], [9], [10] or through acculturation by AMHs [11], [12].

An opposing model has claimed that there is great discontinuity between Neanderthals and modern humans [13], [14] and relates the demise of the Neanderthals to the territorial expansion of AMHs from Africa through the Near East.

The scenarios which have generally been accepted argue that this territorial expansion occurred during a period of great climatic change [15], [16]. According to this hypothesis, the expansion of AMHs, identified primarily through their association with Aurignacian assemblages [13], [17], pushed the Neanderthals associated with Mousterian assemblages toward southern Europe and, in particular, toward the Iberian and Italian peninsulas in the Mediterranean area [11], [18]. This view was reinforced by genetic data which have shown that there is no contribution of Neanderthals to the mitochondrial DNA of *H. sapiens* [19], [20].

During recent years, data collected in Europe that seemed to support this view have been questioned. First, Neanderthal nuclear DNA shows a low level of interbreeding (4%) with sapiens [21]. Furthermore, *H. sapiens* is now associated with local (Uluzzian) so-called "transitional assemblages" at Grotta del Cavallo in the southern Italian peninsula while the human remains were previously thought to be Neanderthals [9], [22]. The presence of AMHs in Grotta del Cavallo has been demonstrated based on the morphological pattern of the enamel on human deciduous teeth, and the age of Uluzzian artifacts associated with the teeth (levels of unit E) has been re-analysed [23]. The new dating shows that the AMHs reached the southern Italian peninsula at around 45–43 ka BP, which is at least 7000 years earlier than was previously supposed. This study indicated the difficulty of advancing a general explanation [11] valid for all of Europe, since the replacement of Neanderthals by AMHs on the Italian peninsula took place earlier [23], [24] and was probably different than that which occurred in Iberia [25]–[27].

**Figure 1:**
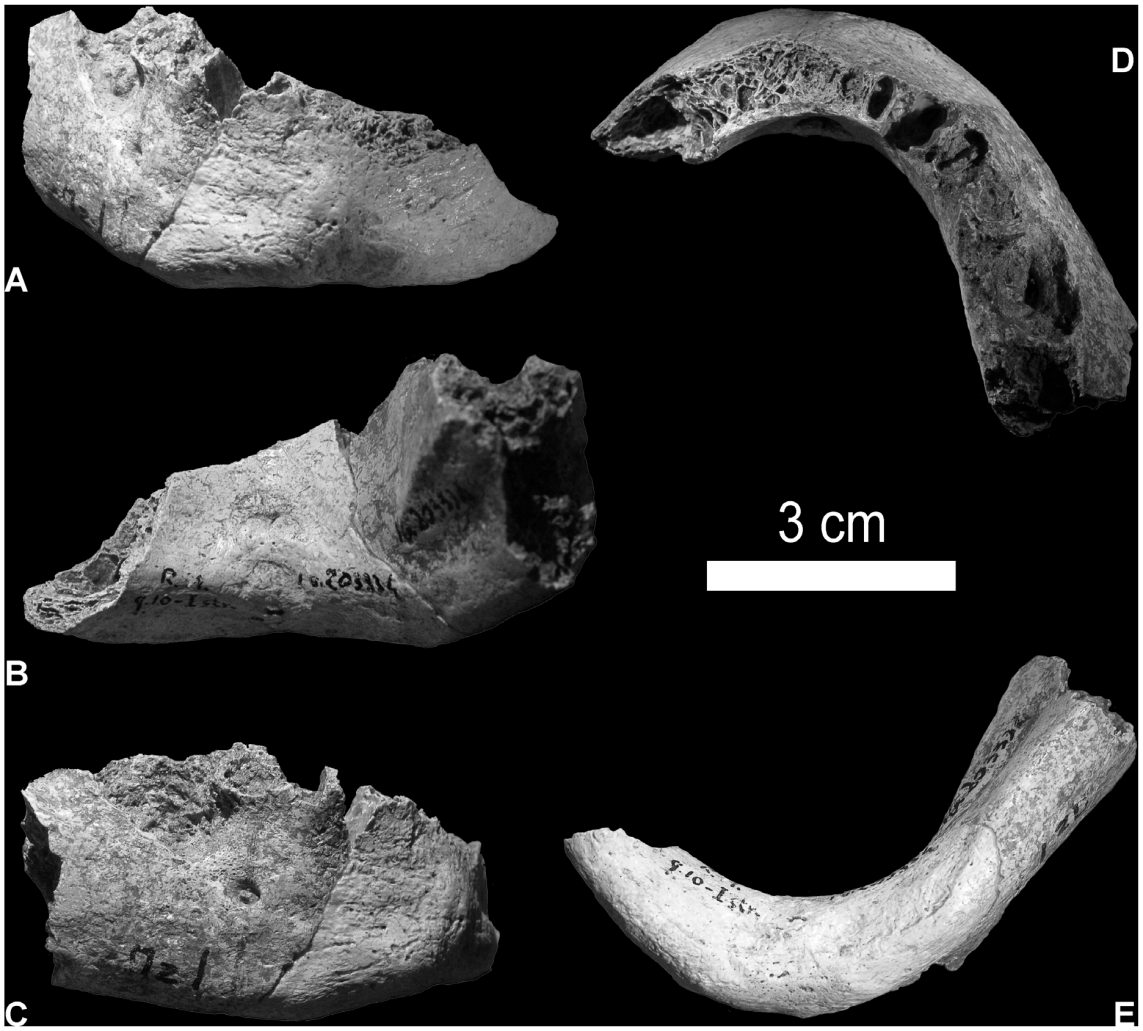
The mandible from Mezzena. Frontal view : A, internal view : B, lateral view: C, superior view : D), inferior view : E.

In this article we examine the morphology of the Mezzena mandible (Figure 1) found in 1957 [28]. We argue that the mandibular morphology of late Italian Neanderthals, in particular the chin, may help us better understand the transition between the two human groups. The study of the human remains of Middle Paleolithic Riparo Mezzena, a rockshelter in the Monti Lessini (Venetian region -NE Italy) associated with Charentian Mousterian lithic assemblages [29], [30] has led to the identification of the only genetically typed Neanderthal of the Italian peninsula (cf. [31]–[33] and this study) and has confirmed through dating that it belongs to a late Neanderthal (i.e. 34.5±655 ka) [30]. Our aim is to re-evaluate the taxonomic affinities of the Mezzena jaw in a wide comparative framework using both comparative morphology and geometric morphometrics analyses. The comparative sample includes mid-Pleistocene fossils, Neanderthals and anatomically modern humans (cf. [34], Tables 1 and S1). This study of the Mezzena mandible shows that the chin region is similar to that of other late Neanderthals which display a much more modern morphology with an incipient mental trigone (e.g. Spy 1, Saint Césaire). In our view, this change in morphology among late Neanderthals reopens the debate on the "more modern like" morphology of late Neanderthals and can lend support to the hypothesis of a certain degree of continuity with AMHs or a possible interbreeding with them.

**Table 1:** Specimens of the study.

Specimens	Hgt mf	Thc mf	RI mf	Hgt M2	Thc M2	RI M2	Labels in Figure 2
Middle Pleistocene specimens							
*H. heidelbergensis*							
**Tighenif 1**	35.5	18.1	51	35.4	22.2	62.6	T1
**Tighenif 2**	31	15	48.4	33	20	60.6	T2
**Tighenif 3**	32.5	19	58.5	-	22.2	-	T3
**Mauer**	33	19.4	58.8	34	20.5	60.2	Ma
**Montmaurin**	28.8	15	52.1	28	159	56.9	Mt
Arago II	33.1	16	53.1	30.5	16.5	54	ArII
Arago XIII	31.2	22	68.7	29	23	79.3	ArXIII
Sima de los Huesos (ATB- 1)	30.2	16.9	55.9	28.9	17.7	61.2	ATB-1
Sima de los Huesos (AT -75)	29.1	15.6	53.6	28.7	16.7	58.1	*
Sima de los Huesos (AT- 250)	31	16.9	54.5	29.5	17.9	60.6	*
Sima de los Huesos (AT -300)	34.3	17.1	49.8	30.1	18.0	59.8	*
Sima de los Huesos (AT-605)	37.1	16.7	45.0	32.9	18.4	55.9	AT-605
Sima de los Huesos (AT -607)	27.1	15.2	56.0	27.2	17.1	62.8	AT-607
**KNM-BK 67**	32.5	14.9	45.9	32.3	20.4	63.1	BK67
**Ehringsdorf F**	25.5	16.5	63.5	27	16	59.3	EhF
Late Pleistocene							
*H. neanderthalensis*							
**Baňolas**	28.9	16	55.5	33	19	57.5	Ba
**La Naulette**	25	14	56	23	17	73.9	Na
**Malarnaud**	24	14	60.4	22	15	68.2	*
**La Chaise BD1**	33	15.1	45.7	30.5	15	50	*
**Krapina G**	30	15	50	28	14.5	51.8	*
**Krapina H**	35	15	42.8	33.5	15	44.8	*
**Krapina J**	33.5	16	47.7	33.2	16.2	48.8	KJ
**Krapina D**	27	13	44.4	-	-	-	*
**Regourdou**	33	16	48.5	32	15	46.8	Reg
**La Quina H5**	34	15	44.1	34	16	47	*
**La Quina H9**	37	16	43.2	-	-	-	QH5
**Arcy-sur-Cure**	38	16.2	42.6	33	19	57.6	*
**La Ferrassie 1**	33	15	45.4	32	14	43.7	LF1
**Spy I**	33	14	42.4	31	16	51.6	Spy1
**Zafarraya**	33.3	16	47.9	32.3	14	43.2	Zaf
Saint Césaire	32	12.6	39.37	28.8	12.7	44.09	StC
**Guattari II**	36	17	47.2	-	-	-	GuII
**Guattari III**	35	14	40	35	16	45.7	GuIII
**Mezzena**	**(34)**	**13**	**(38.3)**	**27.5**	**16.5**	**60**	***Mezzena***
**Tabūn C1**	27.5	15	54.5	26.2	16.2	58	TC1
**Tabūn II** **Amud 1**	42.534	16.415	55.544.1	38.534	1816.5	5848.5	TII*
*H. sapiens*							
**Skhūl V**	(36)	13.2	36.7	(34.5)	13	37.7	SV
**Qafzeh 9**	35	16.6	47.4	29	17	58.6	Q9
**Ohalo II**	30.33	12	39.7	28.54	14.8	51.8	OII
**Cro-Magnon I**	28	13.5	48.2	26.3	13.3	50.6	CMI
**Abri Pataud 1**	27.9	11.7	41.9	28.1	17.2	61.2	AP1

Specimens used in the study. * indicates specimens that were not included in the geometrics morphometric analysis due to the state of preservation of the fossils or to their inaccessibility. Bold types indicate when original fossil was examined. Robustness index (RI) of the mandibular corpus are calculated from the thicknesses (Thc) and the heights (Hgt) at the mental foramen level (mf) and at the M2 level. All measurements have been taken in accord with standard procedures defined by [64] and are derived from [36], with the exception of Zafarraya [39] and the Mezzena mandible (italic, present study).

## Results

### Comparative Morphology

The mandible of Mezzena (IG VR 203334) is incomplete (Figure 1). All of the fractures are old, the two vertical branches and the left side of the mandibular corpus (from P1) are broken. However, the symphyseal region is complete. On its right side the body of the mandible is conserved up to the level of the second molar. No teeth are present in the mandible: most of them were lost *ante-mortem* (Figure 1D). Destruction and pathological remodelling of internal bone in the vicinity of the right premolar and molar teeth was revealed through x-ray and computerized-tomodensitometric examinations. This lesion has been interpreted as subsequent to an infection due to bacterial invasion developed from the right premolar [35].

The Mezzena corpus is somewhat robust. However, this robustness cannot be evaluated with great precision since the alveolar rim is damaged throughout its length, in other words from the level of the first left premolar until the second right molar (Figure 1D). In spite of this damage we have evaluated the robusticity of the mandible. The remaining height is 25 mm at level of the symphysis, 27.4 mm at the level of the second molar (M2) and, where the alveolar rim is less damaged, at the level of the mental foramen, the height is 34 mm. (Table 1). Thus, even if not completely accurate due to damage to the alveolar rim, the height of Mezzena at the mental foramina and at the M2 is situated within the range of the variation recognized for European Neanderthals (mental foramen: v = 36–24 mm, N = 18; second molar: v = 28–33,5 mm, N = 15). The thickness of the symphysis measures 14 mm, whereas it is 13 mm at the level of the mental foramen and 16.5 mm at the level of the M2. Thus, the thickness of the mandibular corpus of Mezzena is also close to those of European Neanderthals (mental foramen: v = 14–16.2 mm; N = 15; second molar: v = 12.7–19 mm; N = 15). The index of robustness (i.e. RI) at the level of the mental foramen is 38.23. Due to damage of the alveolar rim in Mezzena, this index is situated slightly below the lower range of variation of European Neanderthals (v = 60.4–39.37 mm; N = 18; cf. Table 1 and [36]). It is of particular interest to note that of all the European Neanderthal jaws, the Mezzena mandible index of robustness is closest to that of St Césaire (RI = 39.37) and Guattari III (RI = 40) and not far from Spy (RI = 42.4) and Arcy sur Cure (RI = 42.6) (Table 1). The index of robustness at the level of M2 is 60. It is situated in the upper range of variation of classical European Neanderthals (v = 73.9–44; N = 18; cf. Table 1). It should be noted that variation of index of robustness among modern humans is very wide and can include the Neanderthal variation [36].

The external face of the right side of the mandibular corpus is present up to the alveolus of the second molar (Figure 1C). The principal mental foramen is small (with an opening measuring 3.7 mm) and positioned under the second molar. A smaller secondary mental foramen is found below the second premolar and the first molar. This feature is important since it is generally acknowledged that a mental foramen positioned under the first molar or between the first molar and the second premolars is a typically Neanderthal character. Indeed, in 25% of the European Neanderthals the mental foramina are situated between the second premolar and the first molar, in 65% they are located below the first molar and, in the 10%, below the second premolar [36]–[38]. The position of this feature in the Mezzena mandible suggests that it cannot be excluded from the Neanderthals and that the Mezzena jaw was moderately elongated similar to Guattari III, Saint-Césaire, Zafarraya, and Palomas 59 [36], [39], [40]. Additionally, the mental foramina of Mezzena are situated half way up the body of the bone which is usually considered to be an archaic feature [36].

On the lateral surface of the mandibular body of Mezzena (Figure 1C) there is a slight swelling, the *prominentia lateralis*, situated below the location of the second molar and the bone fracture. Near the base of the jaw there is a marginal anterior tubercle (*tuberculum marginalis anterior*). Its size is relatively small. As for the position of the *prominentia lateralis*, the presence of this tubercle is considered to be a diagnosic feature of Neanderthals [36]–[38].

The interior face of the mandibular body of Mezzena (Figure 1B) displays a well-defined oblique internal line or *linea mylohyoidea*, as on Neanderthals. This line is situated, as on Neanderthals, in a lower position than in modern humans. Toward the front and the upper part there is a small *fossa sublingualis*. Due to the state of preservation of the mandible it is not possible to follow this line throughout its length. Thus it is only possible to note toward the back the presence of a *fossa subalveolaris posterior*. The clear relief of the oblique internal line shows that the mandible of Mezzena had powerful mylo-hyoid muscles. In general, this region, as with other parts of the mandible, resembles the morphology found on Neanderthals.

The symphysal region of the Mezzena mandible (i.e. the region delimited by the mesial rim of the canines, Figure 1A) is relatively well-preserved and provides important information. However, the fractures of the alveolar rim do not make it possible to calculate the angles in order to evaluate the inclination of the symphysis. The bone surface displays a swelling in the region of the *trigonum mentale* which is composed of a very small *tuber symphyseo* and of two *tubercula lateralia*, the latter of which are distinctly separated from the inferior margin of the mandible. This surface does not display an *incurvatio mandibulae*. The *incisura submentalis* is present and, in the basal region, it forms a slight concavity in the shape of an arc with a maximum height of 5 mm. In lateral view the symphyseal region does not appear to be concave as among modern humans, nor convex, as among ancient European fossils, but vertical with a slight swelling. Both morphologies of the lateral profile of the symphysis and of the *incisura* are similar to that found among classic Neanderthals, such as Guattari III or Regourdou from France. But this morphology is present in particular among late Neanderthals (e.g. St Césaire, Spy 1, La Ferrassie 1, Las Palomas 59 and Vindija [5], [36]) and to a lesser extent among Neanderthals of the Near East (especially Tabūn II and Amud 1). All these fossils have an incipient *mentum osseum*. On the internal face of the symphysis (Figure 1B), the alveolar rim is severely damaged but the mental spines (*spinae mentales*) can be noticed below the fracture. They are clearly separated as on the Neanderthal La Ferrassie 1. Above these two spines a *foramen spinosum* is clearly visible. Under the upper mental spine there is a very slight half-moon shaped notch comprising the *fossa genioglossa*.

The inferior margin of the mandible (Figure 1D) is very thick and presents visible digastric muscles imprints. They form two digastric fossae which are well delimited and distinct. They are large and ellipsed shaped. At the point of junction of the two fossae there is a marked *crista intergastrica* in front of which is the *trigonum basale* of Toldt. Here too, this region bears a similarity to the Neanderthals.

### Geometric Morphometrics (Shape Analysis)

The M Box test results (M = 207.445, F = 1.150, ddl1 = 110, ddl2 = 3741.902, p = 0.139, Table S3) indicates that the covariance matrices are homogenous, and therefore a linear Discriminant Function Analysis (DFA) is appropriate.

The first discriminant function (F1) of the DFA accounts for 81.0% of the total variance of the discrimination; it separates the three pre-defined groups: *H. sapiens*, *H. neanderthalensis* and mid-Pleistocene specimens which have been previously attributed to *H. heidelbergensis* [35]. This discrimination is supported by a significant Wilks’ lambda value (Wilk’s λ = 0.097, chi-square = 80.504, df = 20, *p*<0.0001, Table S4). Most of the intra-group shape variation is represented along the second function (F2∶19.0% of variance), which, coherently Wilk’s lambda value is less significant (Wilk’s λ = 0.504, chi-square = 23.616, df = 9, *p* = 0.005, Table S4). Nevertheless, these results suggest that the variables can be used to distinguish between the groups. Results of the validation procedure (i.e. cross validation, see Method section) indicate that 78.6% of cross validated grouped specimens were correctly re-classified (i.e. 80% of the modern Humans, 73.3% of the Neanderthals and 83.3% of *H. heidelbergensis*) compare to 95.2% of correct classification during the original DFA procedure (Table S7 and S8). Again, this value suggests that the groups can be distinguished by the DFA.

The linear regressions (PC1: R^2^ = 0.190, *p* = 0.004; PC2: R^2^ = 0.007, *p = *0.591; PC3: R^2^ = 0.005, *p* = 0.653; PC4: R^2^ = 0.203,*p* = 0.002; PC5: R^2^ = 0.016, *p* = 0.425; PC6: R^2^ = 0.006, *p* = 0.630; PC7; R^2^ = 0.013, *p* = 0.468; PC8: R^2^ = 0.005, *p* = 0.657; PC9: R^2^ = 0.047, *p* = 0.164; PC10: R^2^ = 0.013, *p* = 0.459) indicate that centroid size does not significantly impact specimens’ shape (see, Table S6). Thus, differences in shape between specimens are not due to allometry.

**Figure 2:**
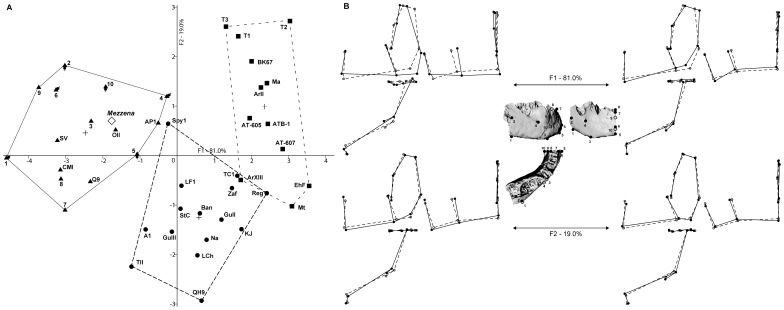
Discriminant Function Analysis based on landmarks data (A) and associated cranial shapes (B). A) Crosses indicate centroïds of each a priori sample. Triangles = modern humans (1–10: Holocene specimens with indication of sex when known, labeled: fossil specimens); circles = Neanderthals; squares = *H. heidelbergensis* sample; diamond = Mezzena included a posteriori in the analysis. Labels descriptions are provided in Table 2. B) The configuration of landmarks is indicated by circles superimposed on views of the Mezzena mandible (full, visible landmarks; empty, landmarks non visible in the current view); shapes in *norma lateralis*(upper left), *norma verticalis*(lower left) and *norma facialis*(right) are portrayed for the extremities of each axis (full lines, shape change; dashed lines, consensus). Modern humans, Neanderthals and middle Pleistocene specimens are discriminated on Function 1. Function 2 mostly shows intra-group morphological variation. The architectural shape of Mezzena is closer to modern human specimens, particularly to Abri Pataud 1. Mezzena’s shape shows also strong similarities with Neanderthals, especially late Neanderthals (e.g. La Ferrassie 1, Spy 1).

The F1 is responsible for most of the dispersion of the cloud of points (Figure 2A). Neanderthals and AMHs groups overlap at the centre of the chart. Late *H. neanderthalensis* specimens (i.e. Saint-Césaire and Spy 1), Near-Eastern Neanderthals (i.e. Tabūn II and Amud 1), as well as the more classic Neanderthal specimens La Ferrassie 1 and Guattari III, tend to be positioned at the left margin of the Neanderthals cloud of points in the overlapping area with AMHs *H. sapiens* fossil specimens are positioned well within of the recent human cloud of points and are not similar in shape to Neanderthals to the exception of Abri Pataud 1. On the contrary, it is two 20^th^ century specimens (i.e. Java 1 and Nigeria 2) which share more similarities in shape with Neanderthals. The mid-Pleistocene specimens are quite homogeneous on F1 where they are segregated from Neanderthals. The cloud of points nevertheless overlaps slightly with Neanderthals. This is mainly due to the position of the Arago XIII and Tighenif 3 mandibles. Most of the dispersion of the cloud of points is observed on F2, and the African and European specimens show strong similarities in their shape, especially the Mauer and KNM-BK 67 specimens.

We can elaborate on the distributions of mandibular shape among hominins if we look at Figure 2B. Extreme shapes for modern humans show the presence of a well-developed chin (*tuber symphyseos*, pogonion, landmark #6), the absence of a *planum alveolare* (planum alveolare, #9) and a relatively gracile mandibular corpus. The Neanderthals are characterized by a more vertical symphyseal profile with no well-developed *tuber symphyseos* (pogonion, #6), a slightly thicker mandibular corpus at the mental foramen level, which is also positioned slightly more posteriorly (foramina mentale, #4). Mid-Pleistocene fossils show a strongly receding symphyseal profile with no chin development (pogonion, #6). The antero-posterior thickness of the symphysis at the level of the dental arch is important (infradentale, #7 and infadentale posterior, #8), and the *planum alveolare* is well-developed (planum alveolare, #9). A wide *incisura submentalis* is present under the lower rim of the symphysis (tuberculus marginalis superius, #3 and gnathion, #5) which is absent in AMHs and weakly developed in Neanderthals.

The position on the scatter plot of our specimen of interest, Mezzena, has been calculated *a posteriori*. Unsurprisingly, the Mezzena mandible does not present any particular affinities with mid-Pleistocene specimens. It is most similar to AMHs being positioned within the *H. sapiens* cloud of points and the DFA classifies the specimen with modern humans (Table S7). Especially its shape is similar to that of Ohalo II and to a lesser extent to the recent modern human specimen China5. However, it should be noted that its position also indicates affinities with some Neanderthal specimens: the late Neanderthal Spy 1 and Saint-Césaire, the Near-East specimens Tabūn II and Amud 1, and to a lesser extent the classic Neanderthals La Ferrassie 1 and Guattari III (Figure 2).

### Genetic Analysis

About 100 mg of bone powder were removed by drilling the bone of the Mezzena mandibular corpus; DNA was extracted in a DNA laboratory exclusively dedicated to ancient DNA work. We performed three different extractions and two different PCR for each extracts. After purification of these PCR products the sequence of the hypervariable region I of mtDNA was divided into seven amplicons but due to high DNA degradation we obtain only the fragment NL 16230 NH16262, in which all sequences were endogenous (Figure S1). All the substitutions observed in the MLS mtDNA jaw (determined between positions 16230 nt to 16262 nt) have been consistently reproduced in different amplifications and in three different extracts.

The comparison with other 30 partial mitochondrial Neanderthal sequences are currently available: Feldhofer 1 and Feldhofer 2 from Germany; Mezmaiskaya from the Russian Caucasus; Okladnikov from Siberia, Russia; Vindija 75, Vindija 77 and Vindija 80 from Croatia; Engis 2 and Scladina from Belgium; La Chapelle-aux-Saints and Rochers-de-Villeneuve from France; El Sidrón 441, El Sidron 1252, El Sidron R011, El Sidron 331c, El Sidron 1327 h, El Sidron 753, El Sidron 1161, El Sidron 763a, El Sidron 566, El Sidron 500, El Sidron 1634, El Sidron 763b, El Sidron 634 and Valdegoba and Cova del Gegant from Spain; Teshik Tash from Uzbekistan plus other five complete mtDNA (in three of them the HVR-I region were previously typed Feldhofer 1 and 2, Vindija, 33.16 previous called V80) and two new samples, El Sidrón 1253 from Spain, and another one from Croatia (Vindija 33.25) (for more details and references see, Table S10).

The new Mezzena mitochondrial sample of the mandible presents a classic Neanderthal motif (16234 T, 16244 A, 16256 A, 16258 G) with the diagnostic transversion 16256 C/A (see, [41] and Table S10). The nucleotides at these sites are very unlikely to reflect contamination, because they were consistently observed in amplicons also showing mutations typical of Neanderthals and not of modern humans. Moreover these substitutions were previously observed in 5 other Neanderthals (Feldhofer 1, Vindija 75, El Sidrón 441, Vindija 80 (33.16)) and in the previous Mezzena (MLS 1) cranial fragments examined [31], [32].

## Discussion

The genetic analysis of the small fragment of hypervariable region 1 of the mtDNA with the well-known diagnostic Neanderthal substitutions (determined between positions 16230 nt to 16262 nt) presents a classic Neanderthal motif (16234 T, 16244 A, 16256 A, 16258 G) with the diagnostic transversion 16256 C/A which classifies the Mezzena mandible as a Neanderthal. These results are further supported by the comparative morphology analysis which shows similarities between the Mezzena mandible and Neanderthals. The symphysis of the Mezzena mandible is very close to the European classic Neanderthals (i.e. Regourdou and Guattari III) and late Neanderthals (Spy, La Ferrassie 1, Saint-Césaire, Vindija and Las Palomas) showing an incipient mental trigone and more vertical symphyses than earlier Neanderthals [5], [36], [40].

However, the pattern is not quite clear, especially considering the results of the DFA analysis where Mezzena mandibular shape is more similar to that of modern humans and is classified as *H. sapiens* (Figure 2, Table S7). The DFA based on geometric morphometrics and Procrustes analysis distinguishes between the three pre-defined groups: mid-Pleistocene fossils (most specimens being attributed to *H. heidelbergensis*, [34]), Neanderthals (*H. neanderthalensis*), and AMHs (*H. sapiens*). The first axis (81.0% of variance) presents a taxonomic-based distribution of the specimens, with *H. sapiens*, *H. neanderthalensis*, and *H. heidelbergensis* being separated along this function. The second axis (19.0% of variance) accounts for most of the intra-group variation.

Nevertheless, overlaps occur between the three groups of specimens. This might be partly due to the chosen landmarks which failed to describe the full mandibular shape. They are concentrated on the symphysis and on the most anterior part of the mandibular corpus. Most of them are type II or III landmarks (see, [42]) which cannot be fully considered as “*discrete anatomical loci that can be recognized as the same loci in all specimens*” ([43]:23). This can explain the relatively high Wilk’s lambda value (see, Table S7). However, the preservation state of the Mezzena mandible does not allow a better description of the full mandibular shape.

This methodological problem does not rule out the fact that the DFA results are taxonomically coherent (i.e. they provide a classification which succeeds in attributing most of the specimens of our sample to their correct alleged species, Figure 2 and Table S7) and support the existence of different species among our data: *H. sapiens* and *H. neanderthalensis* of course, and to a lesser extent *H. heidelbergensis*. Additionally, studies of morphological and metrical variations in AMHs and Neanderthals already show some level of overlap (e.g. [5], [44]–[47]) although the two populations are overall morphologically distinct (e.g. [44], [45], [47]–[49]). Finally, we must keep in mind that most of the Neanderthals diagnostic features have been identified on the mandibular corpus and especially on the ramus (e.g. retromolar space, truncated gonial angle, medial position of intersection between mandibular notch and condyle and deeply excavated pterygoid fossa, see for instance, [36], [38], [50]). Thus, the absence of a ramus and of part of the mandibular corpus on the Mezzena specimen might have artificially enhanced its resemblance to AMHS in the geometric morphometrics analysis.

In this light, we can interpret the position of the Mezzena mandible which stands within the modern human shape space, while presenting strong shape similarities with some Neanderthal specimens. Such a conflicting taxonomical position is not surprising, considering the geological age of the mandible [30]. Indeed, numerous late Neanderthals such as Spy 1, Saint Césaire and the Near-East mandibles Amud 1 and Tabun II possess hints of a chin (i.e. *tuber symphyseo*) though not a true modern human morphology [37], [51]. Late Neanderthals lived in area where AMHs might have been already present [2], [23], [52], while the Levantine fossils are displaying a less derived Neanderthal morphology [35], [36].

Therefore, in our view, this change in morphology of the mandibular chin among the fossils of Mezzena and other late Neanderthals could have been the result of a small degree of interbreeding with AMHs. We must nevertheless keep in mind that this data cannot exclude the possibility that the estimated genetic admixture between *Homo sapiens* and *Homo neanderthalensis* might be due to a sub-structure of an ancient African ancestor of archaic human and present-day human populations [53],or a more complex model recently published [54].

Thus, morphological and geomorphological analyses of the mandible of Riparo Mezzena strongly support the hypothesis of change in morphology on this genetic typed late Mousterian Neanderthal. This study confirms that simple models of abrupt behavioral and phylogenetic transition for this period in Europe should be abandoned, at least in certain geographical areas [5], [44], [55]. In Italy while AMHs with Uluzzian assemblages reached the south of the peninsula at Grotta del Cavallo at around 45–43 ka BP [23] and settled in Northeastern Italy close to the Mezzena rockshelter at the site of Fumane with proto-Aurignacian industries at 41/40 ka cal BP [56], in Riparo Mezzena [30], morphologically and genetically identifiable Neanderthals still making Mousterian industries had not yet disappeared.

## Materials and Methods

### Materials

The fossil sample was selected in order to encompass a large part of morphological variation of the middle and late Pleistocene fossil record. 48 fossils from Africa, Asia, and Europe were studied (middle Pleistocene specimens among which most of the individuals were attributed to *H. heidelbergensis* [34]:18, *H. neanderthalensis*: 24 and *H. sapiens*: 5) (Table 1). Additionally, 10 modern humans from Africa, Europe and Asia (4 Neolithic, 6 extant modern humans among which 3 males, 3 females and 4 non-sexed specimens) were included in the geometric morphometrics analysis in order to: 1- provide a sufficient sample of modern humans spread out over a span of time similar to that of the Neanderthals (i.e. 130,000 years); 2- take into account the margin of error in dating the fossil sample; and 3- test the reliability of the character data set and the statistical method used in the study. The reduce number of modern humans used in the DFA is due to the obligation to respect the hypothesis of equality of the co-variant matrices of the three groups. The three groups must be of roughly equal size (i.e. *H. heidelbergensis* N = 12, *H. neanderthalensis* N = 15, *H. sapiens* N = 15) to be able to interpret the results of the DFA.

### Methods

#### Geometric morphometrics shape analysis

(see, [42]) is based on 10 landmarks (Figure 2, Table 2) chosen to best describe the mandible morphology while taking into consideration the state of preservation of the fossils and especially of the Mezzena specimen. The method follows the protocol described in [57]. We ran a Generalized Procrustes Analysis, a Principal Component Analysis (PCA) based on the procrustes residuals and a Discriminant Function Analysis (DFA) to discriminate three pre-defined groups (*H. sapiens*, *H. neanderthalensis* and *H. heidelbergensis* sensu [34]). The number of variables must be lower than 12 (smallest group number of specimens), thus this analysis uses the first 10 Principle Components (PC) which represent 90.02% of the total variance (Table S2, S4 and S5). The discrimination between these groups is used as a “pattern” to study the Mezzena mandible which is introduced *a posteriori* in the analysis. The Wilks’ lambda statistics (see, Table S4), used to validate the discrimination, necessitates covariance matrices equality of each group which can be tested using a Box’s M test (Table S4). A cross validated classification was then ran. It successively classifies all cases but one to develop a more reliable discriminant function and then categorizes the case that was left out (Table S7 and S8). Additionally, we tested the impact of size on specimen shape modifications in order to identify a possible allometric trend in our data. We used linear regression, which was calculated for each PCs involved in the computation of the discriminant functions when compared to centroid size (Table S6). We used Morphologika 2 v2.5 [58] (APG, ACP, linear regression) and SPSS v11.5 ©SPSS Inc. 1989–2002 (linear Discriminant Function Analysis).

**Table 2:** Landmarks used in for the geometric morphometrics analysis.

n°	Name	Description	type
**1**	**Basal**	point vertically positioned under the centre of the *proeminentia lateralis*	III
**2**	***proeminentia lateralis***	centre of the *proeminentia lateralis*	II
**3**	***tuberculus marginalis superius***	most latero-inferior point of the tubercle	II
**4**	***foramina mentale***	point at the centre of the of the *foramen mentale*	I
**5**	**Gnathion**	most inferior midline point on the mandible	II
**6**	**Pogonion**	most anterior midline point on the chin	II
**7**	**Infradentale**	most anterior midline point between the alveoli of the incisors	II
**8**	**infradentale posterior**	most posterior midline point between the alveoli of the incisors	II
**9**	***planum alveolare***	most posterior midline point of the *planum alveolare*	II
**10**	**Geni**	most posterior midline point of the mental spines	II

Number. name. description and type for each landmark.

#### Genetic analysis, experimental procedures

We performed three different extractions and two different PCRs for each extracts. All of the most stringent protocols and procedures for the analysis of ancient DNA have been followed [59]–[61]. Extraction was performed as described in [62], with UNG treatment [63] in order to minimize postmortem damage. mtDNA sequences were generated by using 60 cycles of PCR and 5 ul of extract. The strong inhibitory effect of the extract required a final 1 to 100 dilution to obtain positive amplifications. Different primer pairs were used, some of them designed to match Neanderthal-specific substitutions (Figure S1, Table S9). The PCR products were cloned using the TOPO TA Cloning kit (Invitrogen), according to the manufacturer’s instructions. Screening of white recombinant colonies was accomplished by PCR. The colonies were transferred into a 30-µl reaction mix (67 mM Tris HCl [pH 8.8], 2 mM MgCl2, 1 µM of each primer, 0.125 µM of each dNTP, and 0.75 U of *Taq* polymerase) containing M13 forward and reverse universal primers. After 5 min at 92°C, 30 cycles of PCR (30 s at 90°C, 1 min at 50°C, 1 min at 72°C) were performed and clones with an insert of the expected size were identified by agarose-gel electrophoresis. After purification of these PCR products with Microcon PCR devices (Amicon), a volume of 1.5 µl was cycle-sequenced, according to the BigDye Terminator kit (Applied Biosystems) supplier’s instructions. The sequence was determined using an Applied BioSystems 3100 DNA sequencer. The hypervariable region I of mtDNA was divided in seven amplicons (L 15995- H16132; L16022- H16095; L 16106- H 16282; NL 16223 -H16385; NL16230- NH16262; L16299-H16400; 3; L16311- H16402) but due to high DNA degradation we obtain only the fragment NL 16230 NH16262, in which all sequences were endogenous (Figure S1). All the substitutions observed in the MLS mtDNA jaw (determined between positions 16230 nt to 16262 nt) have been consistently reproduced in different amplifications and in three different extracts.

## Supporting Information

Figure S1 Sequences of the 31 clones from which the consensus sequence was determined in the MLS Neanderthal jaw sample. The first line reports the human reference sequence (CRS) with the numbering of the nucleotide positions. Second line reports the sequences of primers used. Nucleotides identical to the reference sequence are indicated by dots. Clones are identified by an abbreviation and three numbers: the first number indicates the extraction; the second number indicates the PCR, the third number indicates the amplicon.
(DOCX)

Table S1 Holocene modern humans included in the geometrics morphometric analysis.
(DOCX)

Table S2 Main Principal Components from the procrustes shape analysis. Eigenvalues. percentage of variance and percentage of cumulated variance for each principal component.
(DOC)

Table S3 Discriminant Function Analysis: Box’s M results on the covariance matrices of the three predefined groups. p>0.5, the hypothesis of equality of the covariance matrices is accepted. The covariance matrices of the three groups are considered to be equal.
(DOC)

Table S4 Discriminant Function Analysis: quality of the discrimination. The Wilks’ lambda results validate the discrimination for function 1 at *p*<0.0001. Function 2 is less discriminating (i.e. Wilks’ lambda = 0.504, *p* = 0.005).
(DOC)

Table S5 Discriminant Function Analysis: Principal Component contribution to each discriminant function and coefficient for each function. CP2 and CP1 contribute the most to the first discriminant function while CP3 and 7 contribute the most to the second discriminant function (in bold).
(DOC)

Table S6 Linear regression results for the ten first principal components when compared to centroïde size. The R^2^ values indicate that the linear regression is not a good approximation of the data with a maximum of 19.0% for PC1 and 20.3% PC4 of the data explained by the linear regression. Additionally, Fisher’s tests are not significant
(DOC)

Table S7 Classification of the specimens from the original DFA and from the cross validation procedure. Specimens presented with their original assigned group (i.e. #1: *H. sapiens*, #2: *H. neanderthalensis* and #3: *H. heidelbergensis*) and their predicted group. Discriminant function scores are indicated for each specimen. Incorrectly classified specimens are signalled with **. Note the attribution of the Mezzena mandible to the *H. sapiens* group (#1).
(DOC)

Table S8 Summary of the original classification and of the cross validated classification. 95.2% of original cases are correctly classified and 78.6% of cross validated groups cases are correctly classified. Groups numbers: #1: *H. sapiens*, #2: *H. neanderthalensis* and #3: *H. heidelbergensis.*
(DOC)

Table S9 Primers sequences used in this study. NL, NH: primers designed to match with Neanderthal-specific substitutions.
(DOC)

Table S10 Mitochondrial DNA sequences. MtDNA sequences showing Neanderthal diagnostic positions in HVRI (hypervariable region I).
(DOC)
