# Correction: Possible Interbreeding in Late Italian Neanderthals? New Data from the Mezzena Jaw (Monti Lessini, Verona, Italy)

**DOI:** 10.1371/annotation/b8ec29b1-e71f-4148-baad-1441fca03ed9

**Published:** 2014-01-13

**Authors:** Silvana Condemi, Aurélien Mounier, Paolo Giunti, Martina Lari, David Caramelli, Laura Longo

There are multiple errors in Table S10. Please view the correct Table S10 here: http://plosone.org/corrections/pone.0059781.s011.cn.docx

