# Supplementary material for: Possible Interbreeding in Late Italian Neanderthals? New Data from the Mezzena Jaw (Monti Lessini, Verona, Italy)
Source: PLoS One. 2013 Mar 27;8(3):e59781. doi: 10.1371/journal.pone.0059781 (PMC3609795; doi:10.1371/journal.pone.0059781)
Supplement: Figure S1 — Sequences of the 31 clones from which the consensus sequence was determined in the MLS Neanderthal jaw sample. The first line reports the human reference sequence (CRS) with the numbering of the nucleotide positions. Second line reports the sequences of primers used. Nucleotides identical to the reference sequence are indicated by dots. Clones are identified by an abbreviation and three numbers: the first number indicates the extraction; the second number indicates the PCR, the third number indicates the amplicon. (DOCX) [file pone.0059781.s001.docx]

**Figure S1**

**GTACAGCAATCAACCCTCAACTATCACACATCAACTGCAACTCCAAAGCCACCCCT-CACCCACTAGGATACCAACAAACC**

# 1.1.1 GCACAGCAATCAACCTTCAACTG...T.........A...........A.G...TTACACCCACTAGGATATCAACAAACC

**1.1.2 NL16,230 ...T.........A...........A.G... NH16,262**

**1.1.3 ...T.........A...........A.G...**

**1.1.4 ...T.........A...........A.G...**

**1.1.5 ...T.T.......A.G.........G.G...**

**1.2.6 ...T.........A...........A.G...**

**1.2.7 ...T.........A...........A.G...**

**1.2.8 ...T.....G...A...........A.G...**

**1.2.9 ...T.........A...........A.G...**

**1.2.10 ...T.........A...........A.G...**

**2.1.1 ...T.........A...........A.G...**

**2.1.2 ...T.....................A.G...**

**2.1.3 ...T.........A...........A.G...**

**2.1.4 ...T.........A...........A.G...**

**2.1.5 ...T.........A...........A.G...**

**2.2.6 ...T.........AT....T.....A.G...**

**2.2.7 ...T.........A...........A.G...**

**2.2.8 ...T.........A...........A.G...**

**2.2.9 ...T.........A...........A.G...**

**2.2.10 ...T.........A...........A.G...**

**3.1.1 ...T.........A...........A.G...**

**3.1.1 ...T.........A...........A.G...**

**3.1.2 ...T.....................A.G...**

**3.1.3 ...T.........A...........A.G...**

**3.1.4 ...T.........A...........A.G...**

**3.1.5 ...T.........A...........A.G...**

**3.2.6 ...T.........A...........A.G...**

**3.2.7 ...T.........A.....T.....A.G...**

**3.2.8 ...T.........A...........A.G...**

**3.2.9 ...T.........A...........A.G...**

**3.2.10 ...T.........A...........A.G...**
