# Supplementary material for: Possible Interbreeding in Late Italian Neanderthals? New Data from the Mezzena Jaw (Monti Lessini, Verona, Italy)
Source: PLoS One. 2013 Mar 27;8(3):e59781. doi: 10.1371/journal.pone.0059781 (PMC3609795; doi:10.1371/journal.pone.0059781)
Supplement: Table S1 — Holocene modern humans included in the geometrics morphometric analysis. (DOCX) [file pone.0059781.s002.docx]

**Table S1.**

| **Specimens** | **Chronology** | **Site** | **Labels Figure 2** |
| --- | --- | --- | --- |
| Holocene | | |  |
| **Sahara1** | 6970 ± 130 bp | Hassi-el-Abiod ,Sahara, Mali | **9** |
| **Sahara6** | 6970 ± 130 bp | Hassi-el-Abiod ,Sahara, Mali | **10** |
| **Loisy1** | 3740 ± 120 bp | Loisy-en-Brie, France | **7** |
| **Loisy2** | 3740 ± 120 bp | Loisy-en-Brie, France | **8** |
| **Spita3** | XVII-XIX centuries | Spitalfields, London, United Kingdom | **2** |
| **Roma10** | XIX century | Romania | **1** |
| **China5** | XX century | Chine – Tibet | **3** |
| **Java1** | XX century | Java – Maduras | **4** |
| **Nigeria2** | XX century | Nigeria | **5** |
| **Nigeria10** | XX century | Nigeria | **6** |
