# Supplementary material for: Possible Interbreeding in Late Italian Neanderthals? New Data from the Mezzena Jaw (Monti Lessini, Verona, Italy)
Source: PLoS One. 2013 Mar 27;8(3):e59781. doi: 10.1371/journal.pone.0059781 (PMC3609795; doi:10.1371/journal.pone.0059781)
Supplement: Table S2 — Main Principal Components from the procrustes shape analysis. Eigenvalues. percentage of variance and percentage of cumulated variance for each principal component. (DOC) [file pone.0059781.s003.doc]

**Table S2**

| **PC** | **Eigen values** | **% total variance** | **% cumulated variance** |
| --- | --- | --- | --- |
| **1** | 0.00608 | 31.90 | 31.90 |
| **2** | 0.00295 | 15.46 | 47.37 |
| **3** | 0.00242 | 12.72 | 60.09 |
| **4** | 0.00164 | 8.60 | 68.69 |
| **5** | 0.00105 | 5.51 | 74.20 |
| **6** | 0.00093 | 4.86 | 79.06 |
| **7** | 0.00078 | 4.09 | 83.15 |
| **8** | 0.00061 | 3.20 | 86.35 |
| **9** | 0.00045 | 2.34 | 88.69 |
| **10** | 0.00042 | 2.19 | 90.89 |
