# Supplementary material for: Possible Interbreeding in Late Italian Neanderthals? New Data from the Mezzena Jaw (Monti Lessini, Verona, Italy)
Source: PLoS One. 2013 Mar 27;8(3):e59781. doi: 10.1371/journal.pone.0059781 (PMC3609795; doi:10.1371/journal.pone.0059781)
Supplement: Table S3 — Discriminant Function Analysis: Box’s M results on the covariance matrices of the three predefined groups. p>0.5, the hypothesis of equality of the covariance matrices is accepted. The covariance matrices of the three groups are considered to be equal. (DOC) [file pone.0059781.s004.doc]

**Table S3.**

| **Box’s M** |  | **207.445** |
| --- | --- | --- |
| F |  | 1.150 |
|  | df 1 | 110 |
|  | df 2 | 3741.902 |
|  | p | 0.139 |
