# Supplementary material for: Possible Interbreeding in Late Italian Neanderthals? New Data from the Mezzena Jaw (Monti Lessini, Verona, Italy)
Source: PLoS One. 2013 Mar 27;8(3):e59781. doi: 10.1371/journal.pone.0059781 (PMC3609795; doi:10.1371/journal.pone.0059781)
Supplement: Table S4 — Discriminant Function Analysis: quality of the discrimination. The Wilks’ lambda results validate the discrimination for function 1 at p<0.0001. Function 2 is less discriminating (i.e. Wilks’ lambda = 0.504, p = 0.005). (DOC) [file pone.0059781.s005.doc]

**Table S4.**

| **Functions** | **Wilks’ lambda** | **Chi square** | **df** | **p** | **eigenvalues** | **% of variance** | **Canonical correlation** |
| --- | --- | --- | --- | --- | --- | --- | --- |
| **1** | 0.097 | 80.504 | 20 | >0.0001 | 4.202 | 81.0 | 0.899 |
| **2** | 0.504 | 23.616 | 9 | 0.005 | 0.983 | 19.0 | 0.704 |
