# Supplementary material for: Possible Interbreeding in Late Italian Neanderthals? New Data from the Mezzena Jaw (Monti Lessini, Verona, Italy)
Source: PLoS One. 2013 Mar 27;8(3):e59781. doi: 10.1371/journal.pone.0059781 (PMC3609795; doi:10.1371/journal.pone.0059781)
Supplement: Table S5 — Discriminant Function Analysis: Principal Component contribution to each discriminant function and coefficient for each function. CP2 and CP1 contribute the most to the first discriminant function while CP3 and 7 contribute the most to the second discriminant function (in bold). (DOC) [file pone.0059781.s006.doc]

**Table S5**.

| **PC** | **Contribution to the discriminant functions** | | **Discriminant functions coefficients** | |
| --- | --- | --- | --- | --- |
|  | **Functions** | | **Functions** | |
|  | **1** | **2** | **1** | **2** |
| **1** | **-0.156** | 0.120 | -0.747 | 0.226 |
| **2** | **-0.499** | -0.036 | -1.258 | -0.038 |
| **3** | 0.065 | **-0.479** | 0.261 | -0.759 |
| **4** | 0.156 | 0.180 | 0.730 | 0.261 |
| **5** | 0.072 | -0.115 | 0.406 | -0.233 |
| **6** | -0.030 | -0.234 | -0.141 | -0.442 |
| **7** | 0.136 | 0.238 | 0.398 | 0.375 |
| **8** | 0.049 | -0.175 | 0.252 | -0.336 |
| **9** | 0.055 | 0.211 | 0.313 | 0.362 |
| **10** | -0.045 | **0.346** | -0.185 | 0.596 |
