# Supplementary material for: Possible Interbreeding in Late Italian Neanderthals? New Data from the Mezzena Jaw (Monti Lessini, Verona, Italy)
Source: PLoS One. 2013 Mar 27;8(3):e59781. doi: 10.1371/journal.pone.0059781 (PMC3609795; doi:10.1371/journal.pone.0059781)
Supplement: Table S6 — Linear regression results for the ten first principal components when compared to centroïde size. The R2 values indicate that the linear regression is not a good approximation of the data with a maximum of 19.0% for PC1 and 20.3% PC4 of the data explained by the linear regression. Additionally, Fisher’s tests are not significant (DOC) [file pone.0059781.s007.doc]

**Table S6.**

|  | **R²** | **F** | ***p*** |
| --- | --- | --- | --- |
| **PC1** | 0.190 | 9.599 | 0.004 |
| **PC2** | 0.007 | 0.293 | 0.591 |
| **PC3** | 0.005 | 0.206 | 0.653 |
| **PC4** | 0.203 | 10.432 | 0.002 |
| **PC5** | 0.016 | 0.648 | 0.425 |
| **PC6** | 0.006 | 0.236 | 0.630 |
| **PC7** | 0.013 | 0.535 | 0.468 |
| **PC8** | 0.005 | 0.200 | 0.657 |
| **PC9** | 0.047 | 2.008 | 0.164 |
| **PC10** | 0.013 | 0.560 | 0.459 |
