# Supplementary material for: Possible Interbreeding in Late Italian Neanderthals? New Data from the Mezzena Jaw (Monti Lessini, Verona, Italy)
Source: PLoS One. 2013 Mar 27;8(3):e59781. doi: 10.1371/journal.pone.0059781 (PMC3609795; doi:10.1371/journal.pone.0059781)
Supplement: Table S7 — Classification of the specimens from the original DFA and from the cross validation procedure. Specimens presented with their original assigned group (i.e. #1: H. sapiens, #2: H. neanderthalensis and #3: H. heidelbergensis) and their predicted group. Discriminant function scores are indicated for each specimen. Incorrectly classified specimens are signalled with **. Note the attribution of the Mezzena mandible to the H. sapiens group (#1). (DOC) [file pone.0059781.s008.doc]

**Table S7.**

| **Specimens** | |  | **Highest Group** | |  |  |  | **Discriminant Scores** | |  |
| --- | --- | --- | --- | --- | --- | --- | --- | --- | --- | --- |
| **Original** | | |  |  |  |  |  |  | | |
|  | **Actual Group** | | **Predicted Group** | **p** | **df** | **P(G=g | D=d)** | **Squared Mahalanobis Distance to Centroid** | **F1** | **F2** | |
| T1 | 3 | | 3 | 0.283 | 2 | 0.997 | 2.526 | 1.649 | 2.411 | |
| T2 | 3 | | 3 | 0.178 | 2 | 1 | 3.454 | 3.042 | 2.718 | |
| T3 | 3 | | 3 | 0.157 | 2 | 0.996 | 3.698 | 1.318 | 2.606 | |
| Ma | 3 | | 3 | 0.892 | 2 | 0.993 | 0.228 | 2.422 | 1.463 | |
| ATB-1 | 3 | | 3 | 0.937 | 2 | 0.962 | 0.129 | 2.446 | 0.641 | |
| AT-605 | 3 | | 3 | 0.897 | 2 | 0.932 | 0.217 | 1.956 | 0.756 | |
| AT-607 | 3 | | 3 | 0.615 | 2 | 0.942 | 0.972 | 2.845 | 0.132 | |
| BK67 | 3 | | 3 | 0.619 | 2 | 0.995 | 0.96 | 2.008 | 1.904 | |
| ArII | 3 | | 3 | 0.924 | 2 | 0.989 | 0.159 | 2.26 | 1.376 | |
| ArXIII | 3 | | 2** | 0.398 | 2 | 0.648 | 1.843 | 1.715 | -0.494 | |
| Mt | 3 | | 3 | 0.1 | 2 | 0.656 | 4.603 | 3.096 | -1.025 | |
| EhF | 3 | | 3 | 0.135 | 2 | 0.916 | 4.004 | 3.558 | -0.613 | |
| KJ | 2 | | 2 | 0.503 | 2 | 0.943 | 1.373 | 1.735 | -1.488 | |
| LCh | 2 | | 2 | 0.745 | 2 | 0.997 | 0.59 | 0.547 | -2.017 | |
| TC1 | 2 | | 2 | 0.411 | 2 | 0.641 | 1.779 | 1.621 | -0.407 | |
| TII | 2 | | 2 | 0.117 | 2 | 0.907 | 4.295 | -1.23 | -2.245 | |
| Reg | 2 | | 3** | 0.215 | 2 | 0.508 | 3.07 | 2.417 | -0.762 | |
| Ba | 2 | | 2 | 0.996 | 2 | 0.981 | 0.007 | 0.614 | -1.168 | |
| QH9 | 2 | | 2 | 0.242 | 2 | 1 | 2.84 | 0.659 | -2.934 | |
| LF1 | 2 | | 2 | 0.729 | 2 | 0.951 | 0.631 | 0.117 | -0.609 | |
| A1 | 2 | | 2 | 0.346 | 2 | 0.897 | 2.12 | -0.848 | -1.493 | |
| GuII | 2 | | 2 | 0.831 | 2 | 0.965 | 0.371 | 1.195 | -1.295 | |
| GuIII | 2 | | 2 | 0.736 | 2 | 0.986 | 0.613 | -0.14 | -1.539 | |
| Spy1 | 2 | | 2 | 0.118 | 2 | 0.525 | 4.273 | -0.241 | 0.644 | |
| StC | 2 | | 2 | 0.871 | 2 | 0.979 | 0.276 | 0.092 | -1.074 | |
| Zaf | 2 | | 2 | 0.564 | 2 | 0.801 | 1.145 | 1.479 | -0.658 | |
| ***Mezzena*** | ***ungrouped*** | | ***1*** | ***0.754*** | ***2*** | ***0.987*** | ***0.565*** | ***-1.763*** | ***0.701*** | |
| Na | 2 | | 2 | 0.884 | 2 | 0.992 | 0.248 | 0.795 | -1.702 | |
| Q9 | 1 | | 1 | 0.61 | 2 | 0.983 | 0.987 | -2.338 | -0.526 | |
| SV | 1 | | 1 | 0.746 | 2 | 1 | 0.586 | -3.225 | 0.306 | |
| CMI | 1 | | 1 | 0.605 | 2 | 0.999 | 1.004 | -3.144 | -0.288 | |
| AP1 | 1 | | 1 | 0.141 | 2 | 0.582 | 3.914 | -0.507 | 0.659 | |
| OII | 1 | | 1 | 0.714 | 2 | 0.977 | 0.674 | -1.656 | 0.52 | |
| Sahara1 | 1 | | 1 | 0.298 | 2 | 1 | 2.421 | -3.729 | 1.379 | |
| Sahara6 | 1 | | 1 | 0.557 | 2 | 0.998 | 1.172 | -1.924 | 1.39 | |
| Loisy1 | 1 | | 1 | 0.256 | 2 | 0.994 | 2.727 | -3.02 | -1.101 | |
| Loisy2 | 1 | | 1 | 0.523 | 2 | 0.999 | 1.296 | -3.13 | -0.473 | |
| Spita3 | 1 | | 1 | 0.341 | 2 | 1 | 2.154 | -3.025 | 1.819 | |
| Rouma10 | 1 | | 1 | 0.113 | 2 | 1 | 4.366 | -4.498 | -0.063 | |
| China5 | 1 | | 1 | 0.96 | 2 | 0.998 | 0.082 | -2.316 | 0.696 | |
| Java1 | 1 | | 1 | 0.068 | 2 | 0.533 | 5.391 | -0.274 | 1.197 | |
| Nigeria2 | 1 | | 1 | 0.343 | 2 | 0.754 | 2.139 | -1.077 | 0.027 | |
| Nigeria10 | 1 | | 1 | 0.503 | 2 | 1 | 1.375 | -3.262 | 1.328 | |
| **Cross validated** | | |  |  |  |  |  |  |  | |
| T1 | 3 | | 3 | 0.061 | 10 | 0.992 | 17.662 |  |  | |
| T2 | 3 | | 3 | 0.01 | 10 | 1 | 23.156 |  |  | |
| T3 | 3 | | 3 | 0.106 | 10 | 0.984 | 15.789 |  |  | |
| Ma | 3 | | 3 | 0.995 | 10 | 0.992 | 2.117 |  |  | |
| ATB-1 | 3 | | 3 | 0.521 | 10 | 0.907 | 9.114 |  |  | |
| AT-605 | 3 | | 3 | 0.985 | 10 | 0.905 | 2.861 |  |  | |
| AT-607 | 3 | | 3 | 0.903 | 10 | 0.903 | 4.811 |  |  | |
| BK67 | 3 | | 3 | 0.009 | 10 | 0.983 | 23.465 |  |  | |
| ArII | 3 | | 3 | 0.361 | 10 | 0.977 | 10.961 |  |  | |
| ArXIII | 3 | | 2** | 0.884 | 10 | 0.858 | 5.099 |  |  | |
| Mt | 3 | | 2** | 0.113 | 10 | 0.9 | 15.553 |  |  | |
| EhF | 3 | | 3 | 0.173 | 10 | 0.682 | 13.998 |  |  | |
| KJ | 2 | | 2 | 0.822 | 10 | 0.898 | 5.914 |  |  | |
| LCh | 2 | | 2 | 0 | 10 | 0.984 | 62.098 |  |  | |
| TC1 | 2 | | 3** | 0.238 | 10 | 0.848 | 12.746 |  |  | |
| TII | 2 | | 1** | 0.001 | 10 | 0.942 | 28.676 |  |  | |
| Reg | 2 | | 3** | 0.127 | 10 | 0.968 | 15.15 |  |  | |
| Ba | 2 | | 2 | 0.007 | 10 | 0.897 | 24.173 |  |  | |
| QH9 | 2 | | 2 | 0.148 | 10 | 1 | 14.587 |  |  | |
| LF1 | 2 | | 2 | 0.259 | 10 | 0.836 | 12.399 |  |  | |
| A1 | 2 | | 2 | 0.718 | 10 | 0.773 | 7.083 |  |  | |
| GuII | 2 | | 2 | 0.77 | 10 | 0.936 | 6.522 |  |  | |
| GuIII | 2 | | 2 | 0.467 | 10 | 0.963 | 9.699 |  |  | |
| Spy1 | 2 | | 1** | 0.23 | 10 | 0.826 | 12.889 |  |  | |
| StC | 2 | | 2 | 0.985 | 10 | 0.971 | 2.835 |  |  | |
| Zaf | 2 | | 2 | 0.754 | 10 | 0.645 | 6.692 |  |  | |
| Na | 2 | | 2 | 0.188 | 10 | 0.984 | 13.685 |  |  | |
| Q9 | 1 | | 1 | 0.451 | 10 | 0.947 | 9.878 |  |  | |
| SV | 1 | | 1 | 0.018 | 10 | 1 | 21.517 |  |  | |
| CMI | 1 | | 1 | 0.048 | 10 | 0.997 | 18.411 |  |  | |
| AP1 | 1 | | 2** | 0.372 | 10 | 0.743 | 10.815 |  |  | |
| OII | 1 | | 1 | 0.14 | 10 | 0.874 | 14.781 |  |  | |
| Sahara1 | 1 | | 1 | 0.072 | 10 | 1 | 17.12 |  |  | |
| Sahara6 | 1 | | 1 | 0.53 | 10 | 0.994 | 9.02 |  |  | |
| Loisy1 | 1 | | 1 | 0.902 | 10 | 0.99 | 4.839 |  |  | |
| Loisy2 | 1 | | 1 | 0.034 | 10 | 0.995 | 19.56 |  |  | |
| Spita3 | 1 | | 1 | 0.004 | 10 | 1 | 25.717 |  |  | |
| Rouma10 | 1 | | 1 | 0.302 | 10 | 1 | 11.748 |  |  | |
| China5 | 1 | | 1 | 0.689 | 10 | 0.996 | 7.383 |  |  | |
| Java1 | 1 | | 3** | 0.27 | 10 | 0.532 | 12.234 |  |  | |
| Nigeria2 | 1 | | 2** | 0.063 | 10 | 0.909 | 17.542 |  |  | |
| Nigeria10 | 1 | | 1 | 0 | 10 | 1 | 34.626 |  |  | |
