# Supplementary material for: Possible Interbreeding in Late Italian Neanderthals? New Data from the Mezzena Jaw (Monti Lessini, Verona, Italy)
Source: PLoS One. 2013 Mar 27;8(3):e59781. doi: 10.1371/journal.pone.0059781 (PMC3609795; doi:10.1371/journal.pone.0059781)
Supplement: Table S8 — Summary of the original classification and of the cross validated classification. 95.2% of original cases are correctly classified and 78.6% of cross validated groups cases are correctly classified. Groups numbers: #1: H. sapiens, #2: H. neanderthalensis and #3: H. heidelbergensis. (DOC) [file pone.0059781.s009.doc]

**Table S8.**

|  |  | **Group** | **Predicted Group Membership** | | | **Total** |
| --- | --- | --- | --- | --- | --- | --- |
|  |  |  | **1** | **2** | **3** |  |
| **Original** | Count | 1 | 15 | 0 | 0 | 15 |
|  |  | 2 | 0 | 14 | 1 | 15 |
|  |  | 3 | 0 | 1 | 11 | 12 |
|  |  | Ungrouped cases | 1 | 0 | 0 | 1 |
|  | % | 1 | 100 | 0 | 0 | 100 |
|  |  | 2 | 0 | 93.3 | 6.7 | 100 |
|  |  | 3 | 0 | 8.3 | 91.7 | 100 |
|  |  | Ungrouped cases | 100 | 0 | 0 | 100 |
| **Cross-validated** | Count | 1 | 12 | 2 | 1 | 15 |
|  |  | 2 | 2 | 11 | 2 | 15 |
|  |  | 3 | 0 | 2 | 10 | 12 |
|  | % | 1 | 80 | 13.3 | 6.7 | 100 |
|  |  | 2 | 13.3 | 73.3 | 13.3 | 100 |
|  |  | 3 | 0 | 16.7 | 83.3 | 100 |
