# Supplementary material for: Possible Interbreeding in Late Italian Neanderthals? New Data from the Mezzena Jaw (Monti Lessini, Verona, Italy)
Source: PLoS One. 2013 Mar 27;8(3):e59781. doi: 10.1371/journal.pone.0059781 (PMC3609795; doi:10.1371/journal.pone.0059781)
Supplement: Table S9 — Primers sequences used in this study. NL, NH: primers designed to match with Neanderthal-specific substitutions. (DOC) [file pone.0059781.s010.doc]

**Table S9.**

| **Primers** | **Fragment Length (primers included)** |
| --- | --- |
| L15995 CCACCATTAGCACCCAAAG  NH 16132 TACCATAATTACTTGACTACC | 180 |
| L16022 TACCATAATTACTTGACTACC  H16095 TACCATAATTACTTGACTACC | 113 |
| L16106 TACCATAATTACTTGACTACC  H16282 CAAACCTACCCACCCTTACC | 217 |
| NL 16223 CAAACCTACCCACCCTTACC  NH16385 AATAGGGGTCCCTTGACCACCA | 204 |
| L 16299 CCAACAAACCTACCCACCCTTA  NH16400 ATTGATTTCACGGAGGATGG | 143 |
| NL 16311 CCAACAAACCTACCCACCCTTA  H16402 GATTTCACGGAGGATGGTG | 132 |
| NL16,230 GCACAGCAATCAACCTTCAACTG  NH16,262 TTACACCCACTAGGATATCAACAAACC | 82 |
