# Supplementary material for: Possible Interbreeding in Late Italian Neanderthals? New Data from the Mezzena Jaw (Monti Lessini, Verona, Italy)
Source: PLoS One. 2013 Mar 27;8(3):e59781. doi: 10.1371/journal.pone.0059781 (PMC3609795; doi:10.1371/journal.pone.0059781)
Supplement: Table S10 — Mitochondrial DNA sequences. MtDNA sequences showing Neanderthal diagnostic positions in HVRI (hypervariable region I). (DOC) [file pone.0059781.s011.doc]

**Table S10.**

| **Fossil specimen** | **Country** | **mtDNA region** | **Length (bp)** | **Diagnostic Neanderthals trasversion in HVR1 according to** | **Reference** |
| --- | --- | --- | --- | --- | --- |
| Feldhofer 1 | Germany | Complete mtDNA | 16565 | 16139 A/T  16256 C/A  Insertion 16263 A |  |
| Feldhofer 2 | Germany | Complete mtDNA | 16565 | 16139 A/T  16256 C/A  Insertion 16263 A |  |
| Mezmaiskaya | Russia | Complete mtDNA | 16565 | 16139 A/T  16256 C/A  Insertion 16263 A |  |
| Vindija 75 | Croatia | HVR1 | 357 | 16139 A/T  16256 C/A  Insertion 16263 A |  |
| Vindija 77 | Croatia | HVR1 | 31 | 16256 C/A |  |
| Vindija 80 (33.16) | Croatia | Complete mtDNA | 31 | 16139 A/T  16256 C/A  Insertion 16263 A |  |
| Vindija 33.25 |  | Complete mtDNA | 16565 | 16139 A/T  16256 C/A  Insertion 16263 A |  |
| Engis 2 | Belgium | HVR1 | 31 | 16256 C/A |  |
| Le Chapelle-aux-Saint | France | HVR1 | 31 | 16256 C/A |  |
| Rochers de Villenueve | France | HVR1 | 31 | 16256 C/A |  |
| Scladina | Belgium | HVR1 | 123 | 16256 C/A |  |
| Monte Lessini | Italy | HVR1 | 378 | 16139 A/T  16256 C/A  Insertion 16263 A |  |
| Monte Lessini Mandibula | Italy | HVR1 | 31 | 16256 C/A | This paper |
| El Sidron SD-441 | Spain | HVR1 | 47 | 16256 C/A |  |
| El Sidron SD-1252 | Spain | HVR1 | 303 | 16139 A/T  16256 C/A  Insertion 16263 A |  |
| EL Sidron 1253 | Spain | Complete MtDNA | 16565 | 16139 A/T  16256 C/A  Insertion 16263 A |  |
| Valdegoba | Spain | HVR1 | 303 | 16139 A/T  16256 C/A  Insertion 16263 A |  |
| Teshik Tash | Uzbekistan | HVR1 | 190 | 16139 A/T  16256 C/A  Insertion 16263 A |  |
| Okladnikov | Russia | HVR1 | 348 | 16139 A/T  16256 C/A  Insertion 16263 A |  |

**Table S10 references**

1. Krause J, Orlando L, Serre D, Viola B, Prufer K, et al. (2007) Neanderthals in central Asia and Siberia. Nature 449: 902-904.

2. Krings M, Stone A, Schmitz RW, Krainitzki H, Stoneking M, et al. (1997) Neandertal DNA sequences and the origin of modern humans. Cell 90: 19-30.

3. Briggs AW, Good JM, Green RE, Krause J, Maricic T, et al. (2009) Targeted retrieval and analysis of five Neandertal mtDNA genomes. Science 325: 318-321.

4. Schmitz RW, Serre D, Bonani G, Feine S, Hillgruber F, et al. (2002) The Neandertal type site revisited: interdisciplinary investigations of skeletal remains from the Neander Valley, Germany. Proc Natl Acad Sci U S A 99: 13342-13347.

5. Ovchinnikov IV, Götherström A, Romanova GP, Kharitonov VM, Lidén K, et al. (2000) Molecular analysis of Neanderthal DNA from the northern Caucasus. Nature 404: 490-493.

6. Krings M, Capelli C, Tschentscher F, Geisert H, Meyer S, et al. (2000) A view of Neandertal genetic diversity. Nat Genet 26: 144-146.

7. Serre D, Langaney A, Chech M, Teschler-Nicola M, Paunovic M, et al. (2004) No evidence of Neandertal mtDNA contribution to early modern humans. PLoS Biol 2: E57.

8. Green RE, Malaspinas AS, Krause J, Briggs AW, Johnson PL, et al. (2008) A complete Neandertal mitochondrial genome sequence determined by high-throughput sequencing. Cell 134: 416-426.

9. Beauval C, Maureille B, Lacrampe-Cuyaubere F, Serre D, Peressinotto D, et al. (2005) A late Neandertal femur from Les Rochers-de-Villeneuve, France. Proc Natl Acad Sci U S A 102: 7085-7090.

10. Orlando L, Darlu P, Toussaint M, Bonjean D, Otte M, et al. (2006) Revisiting Neandertal diversity with a 100,000 year old mtDNA sequence. Curr Biol 16: R400-402.

11. Caramelli D, Lalueza-Fox C, Condemi S, Longo L, Milani L, et al. (2006) A highly divergent mtDNA sequence in a Neandertal individual from Italy. Curr Biol 16: R630-632.

12. Lalueza-Fox C, Sampietro ML, Caramelli D, Puder Y, Lari M, et al. (2005) Neandertal evolutionary genetics: mitochondrial DNA data from the iberian peninsula. Mol Biol Evol 22: 1077-1081.

13. Lalueza-Fox C, Krause J, Caramelli D, Catalano G, Milani L, et al. (2006) Mitochondrial DNA of an Iberian Neandertal suggests a population affinity with other European Neandertals. Curr Biol 16: R629-630.

14. Dalen L, Orlando L, Shapiro B, Brandstrom-Durling M, Quam R, et al. (2012) Partial genetic turnover in neandertals: continuity in the East and population replacement in the West. Mol Biol Evol 29: 1893-1897.
